# Ability and Trait Emotional Intelligence: Do They Contribute to the Explanation of Prosocial Behaviour?

**DOI:** 10.3390/ejihpe13060073

**Published:** 2023-06-02

**Authors:** Ana Babić Čikeš, Jasmina Tomašić Humer

**Affiliations:** Faculty of Humanities and Social Sciences Osijek Croatia, 31000 Osijek, Croatia

**Keywords:** emotional intelligence, empathy, prosocial behaviour, tests, self-reports, university students

## Abstract

Previous research on ability emotional intelligence (EI) has shown that EI positively contributes to different positive life outcomes. However, the role of EI abilities in prosocial behaviour (PSB) has not been sufficiently investigated. The aim of this study is to investigate the relationships between EI abilities measured by tests and self-reports, empathy and PSB in the student population. A total of N = 331 university students completed a sociodemographic questionnaire, two EI tests, and self-report measures of EI, cognitive empathy, emotional reactivity and PSB. Of all EI measures, only self-reports correlated with PSB. Cognitive and emotional empathy were also related to PSB. Hierarchical regression analysis showed that self-assessed EI, cognitive empathy and emotional reactivity were predictors of PSB. Cognitive empathy and emotional reactivity also mediated the relationship between self-assessed EI and PSB. The results showed that for the prediction of PSB, it is important how a person evaluates his emotional abilities, and not what the actual level of these abilities is. Furthermore, people with higher self-estimated EI behave prosocially more often because they experience empathy to a greater extent, both cognitively and emotionally.

## 1. Introduction

Previous research on ability emotional intelligence (EI) has shown that it positively contributes to various, and for most people desired, life outcomes, such as academic and work success, quality of social relationships, well-being and health [1,2]. However, some authors point to the possibility that emotionally intelligent individuals use their abilities to the detriment of others, for the purpose of manipulation and achieving morally questionable goals [3,4]. In today’s world, which is characterised by many crises, the actions of people who contribute to the well-being of humanity and the community without expecting immediate benefits are very important. One of the prerequisites for such actions could be the ability to understand the situations in which other people find themselves, as well as their own emotions and emotional reactions [5]. Our reactions to other people’s suffering and the constructive use of emotions could be factors that motivate us to act prosocially. Although the relation between person’s empathy and their prosocial behaviour (PSB) has been confirmed [6], the role of EI abilities in PSB has not been sufficiently investigated. The relationship between self-reported EI and PSB has received considerable attention in recent research [7,8,9,10], but studies on the relationship between ability EI and PSB are quite rare [11]. Understanding relationship between EI and PSB could be particularly important for young people who will have the opportunity to create a world that will be able to respond to the challenges of today. This study aims to fill the gap in research on the relationship between EI abilities and PSB, and to compare the roles of ability and trait EI in predicting PSB. Therefore, the aim of this study is to investigate the relationships between ability and trait EI, empathy and PSB in the student population.

This research is based on the best-known ability EI model [12,13]. EI has been defined as (a) the ability to observe, evaluate and express emotions; (b) ability to perceive and generate emotions that facilitate thinking; (c) ability to understand emotions; (d) ability to regulate one’s own and other’s emotions in order to promote emotional and intellectual development [13]. The aforementioned abilities are measured with different types of measures (mainly tests and self-report questionnaires), and when measured in different ways, they represent different constructs. EI measured with ability tests represents one of the types of intelligence [13], while EI measured by self-report questionnaires represents a construct of emotional self-efficacy [14]. Considering that EI tests measure a person’s ability to successfully solve problems related to emotions, and self-reports measure how individuals assess their emotional abilities, they have different relationships with other constructs [15].

PSB refers to actions that are intended to benefit other people other than ourselves and include sharing, helping, confronting and cooperating [16]. It is often associated with the concept of altruism [17], but it may or may not be motivated by altruism [16]. The presence of PSB is explained by various dispositional, environmental and situational factors. Of the situational factors, we can mention various external and internal rewards [18] which are the consequences of such behaviour; of the environmental factors, there are social norms related to PSB [19]; and of dispositional factors, we can mention gender [20] and personal values [21]. To produce PSB, an individual has to pay attention to the needs of another person, determine the intention to help and link their intention to behaviour [22].

One of the constructs that is often associated with PSB is empathy [23]. Empathy refers to resonating with the emotional state of another person and to understanding the position of that person based on the imagined or perceived situation in which he or she is [24] (p. 107). As it can be seen from the definition, empathy has both cognitive and emotional components [25]. According to the altruism–empathy hypothesis, understanding and experiencing the emotions of people in need encourages a person to help [26]. In different words, altruistic (one form of the PSB) behaviour can occur if it is preceded by empathic care for others. Research has confirmed the positive association of empathy with PSB [27,28] as well as with altruistic behaviour [29,30]. There are not many studies on relations between cognitive and emotional aspects of empathy, separately, with PSB, but existing research confirms that both aspects are related to PSB [31].

People with a higher level of EI should be able to experience more empathy [32], given that the perception and understanding of emotions is an integral part of EI. Research shows that correlations of EI abilities with empathy are mostly positive and low, and are higher with self-reports than with ability tests [32,33,34]. Considering the conceptual link between the constructs of EI and empathy, it can be expected that EI abilities will also be related to PSB [35]. Akamatsu and Gherghel [3] proposed that EI in the presence of empathy and morality leads to PSB, but in the presence of Machiavellianism leads to antisocial behaviour. Several studies have confirmed that self-assessed EI is related to PSB in high school students [8,35] and college students [5,22,36], but there is not much research on the relationship between emotional abilities and PSB. Lopes et colleagues’ study on 76 college students [11] showed that emotion-regulation abilities measured by test (fourth level of the EI ability model) are related to peers’ nominations of PSB. One research study found that the ability of recognising fear in faces (first level) was related to PSB in experimental settings [37]. More empirical data on the relationship between EI abilities and PSB, including empathy, are needed to provide a clearer picture of the relationships between these variables, especially because most of the previous research has investigated the relationship between self-assessed EI and PSB. It could be the basis for potential practical work in the field of PSB. In this research, we will test the part of Akamatsu and Gherghel’s model related to PSB, including two types of EI measures, tests and self-reports.

In this paper, we deal with the ability to understand (third level) and manage (fourth level) emotions. The ability to understand emotions should contribute to a person’s understanding of another’s situations, and the ability to manage one’s own and others’ emotions should contribute to finding the best way to help another person with the capacities we have.

This paper investigates the interrelationships of EI, measured by tests and self-reports, with emotional and cognitive empathy and PSB. The aim is to examine whether PSB can be predicted based on EI abilities, self-assessed EI, and cognitive and emotional empathy. Based on previous results, it is expected that self-assessed EI, as well as cognitive and emotional empathy, will be positive predictors of PSB. The relationship between EI tests and PSB has not previously been sufficiently investigated. Considering that self-reported EI and empathy, constructs that are conceptually related to EI, are positively related to PSB, we would assume that EI abilities measured by tests should also positively contribute to PSB. However, results on EI tests and EI questionnaires have different relationships with different constructs, as well as with empathy, so they could have different relations with PSB as well. Considering that EI tests have weaker correlations with self-report measures, in comparison with self-reported EI [38], we hypothesise that self-reported EI will have higher correlations with PSB. Furthermore, we test whether cognitive and emotional empathy mediates the relationship between two forms of EI (abilities and self-assessed) and PSB, as it is proposed by Akamatsu and Gherghel [3]. It is expected that cognitive and emotional empathy are mediators of the relationship between ability EI and PSB and self-assessed EI and PSB.

## 2. Methods

### 2.1. Participants

A total of N = 331 university students (from different faculties and different study years) participated in the study. A convenient sampling method was used. A total of 32 participants were excluded from data analysis due to negligent or incomplete completion of two or more questionnaires required for this research. The final sample consisted of 205 women and 94 men. Average age was 20.91 years (SD = 1.79, range: 19–29). Of the different faculties, 36.7% of the participants attended the Faculty of Civil Engineering, 35.3% attended the Faculty of Education and 28% of the participants attended the Faculty of Law.

### 2.2. Instruments

The sociodemographic questionnaire was used to examine the sociodemographic characteristics (gender, age and faculty) of the participants.

To measure EI abilities, we applied two ability tests, one intended to measure the ability to understand emotions (Analysis of Emotions Test; TAE) [39], and another intended to measure the ability to manage emotions (Emotion Management Test; TUE) [40]. The EI questionnaire (ESCQ) was used to measure self-assessed EI [41].

The Analysis of Emotions Test (TAE) [39] was used to measure the ability to understand emotions. It consists of 25 tasks in which participants are asked to choose two out of six emotions that are always present in a presented complex emotion and two out of six emotions that are never present in a same presented complex emotion. The performance of an individual task is evaluated as the number of correctly identified elementary emotions and can vary in the range from 0 to 4. The total score in TAE is formed as a simple linear combination of performance in all 25 tasks and can theoretically vary between 0 and 100. A higher score indicates a better ability to understand emotions. The test was used on college student and adult samples and showed satisfactory psychometric properties [39,40,41,42]. Cronbach α coefficients of this and other instruments in this research are presented in Table 1.

The Emotion Management Test (TUE) [40] was used to measure the ability to manage emotions in adults. The first version of the test was used to measure this ability in early adolescents [43,44]. The version of the test used in this research consists of thirteen hypothetical problem situations in which different emotions appear. For each situation, four possible actions of a person in that situation are presented, and they differ in how useful they are for the person to mitigate the negative or maintain the positive emotions. The test consists of a total of 52 items (13 problem situations × 4 presented actions). The task of the participants is to assess how useful or harmful each presented action is on a scale from −3 (very harmful) to 3 (very useful). The accuracy of the answers is determined according to the expert’s criteria, whereby the correct answer is given with two points, the adjacent answers with one point, and all other answers with zero points. The total score in the test is calculated as the sum of the points on all items, and a higher score indicates better management of emotions. Previous application of the test showed that it has satisfactory reliability and validity [40].

The Emotional Skills and Competence Questionnaire (ESCQ) [41] was used for self-assessment of EI. It contains 45 items divided into three subscales, the scale of ability to perceive and understand emotions, the scale of abilities to express and label emotions and the scale of managing emotions. The task of the participants is to assess the presence of described abilities in their behaviour on a Likert-type scale from 1 (not at all) to 5 (absolutely yes). The test has been widely used in different countries and different samples [45]. A factor analysis of the questionnaire confirms the three-factor structure, but subscales are moderately positively correlated, and the use of total score is suggested by the author [45]. The total score is formed as a simple linear combination of all items’ scores and represents the measure of general emotional competence.

To measure PSB, the altruism scale [46] was used. It consists of 17 statements, most of which describe situations of helping others with a certain sacrifice, while neglecting one’s own interests. Each item is answered on a five-point Likert-type scale that describes how often a certain behaviour occurred, where 0 means never, and 4 means very often. The total score is calculated as a sum of assessments of each statement. The theoretical maximum score is 68, and a higher score on the scale indicates a greater tendency to PSB. The test showed satisfactory psychometric properties in previous research [46,47].

A Croatian short version of the Empathy Quotient (EQ-28) [48,49] was used to measure empathy. The questionnaire consists of 28 statements for which the participants should mark the level of agreement or disagreement on a Likert-type scale from 1 (completely disagree) to 4 (completely agree). The total score is calculated as a sum of assessments of each statement. The short version of the empathy quotient consists of three subscales, namely cognitive empathy, emotional reactivity and social skills. The factor analysis of the results on the Croatian sample confirmed the three-factor solution with the factors proposed by the authors of the original version [49]. The cognitive empathy and emotional reactivity subscales were used in this study, and they showed very good psychometric properties in previous research [49].

### 2.3. Procedure

The research was conducted at three different faculties of the J.J. Strossmayer University of Osijek. The approval of the Ethics Committee was obtained before conducting the research. Prior to data collection, faculty deans were asked for informed consent for data collection at their respective faculties. The collection of data was conducted during class, and filling out the instruments took approximately 45 min. The general purpose and procedure of the research were explained verbally to the participants. It was emphasised that the research was anonymous and voluntary, that participants could withdraw from participation at any time, and that the obtained results would be used exclusively for research purposes.

## 3. Results

The data were processed in the Statistical Package for Social Sciences (SPSS). First, we calculated the descriptive statistics of the variables (M, SD, Min, Max, Theoretical Range and Cronbach α) and their intercorrelations. After that, a hierarchical regression analysis for explanation of PSB variance was conducted. In the last step, a mediation analysis was conducted, to analyse whether empathy (emotional reactivity and cognitive empathy) mediated the relationship between self-assessed EI and PSB. A power analysis for a linear multiple regression [50] indicated that the minimum sample size yielded a statistical power of at least 0.95 with an alpha of 0.05 and an effect size of d = 0.15 is 146. Table 1 contains descriptive data for all variables used in this research, as well as values of the internal consistency coefficients (Cronbach’s alphas).

The mean values and standard deviations of TAE and ESCQ are in line with previous results on students’ sample [42], while the same coefficients of TUE are in line with those obtained in the study by Babić Čikeš and colleagues [40] on the sample of adults. The values of the cognitive empathy and emotional reactivity are in accordance with the values from the study by Wertag and Hanzec [49] on a Croatian sample of adult non-students. The values of PSB are similar to that obtained in the research conducted by Raboteg-Šarić [47] on a sample of adolescents.

In Table 2, the intercorrelations of the analysed variables are presented. Girls have higher results on all measures except ESCQ and PSB. EI tests are positively and moderately intercorrelated. TAE results do not correlate significantly with ESCQ results, while TUE results are in low correlation with ESCQ results. Furthermore, EI tests were positively related to emotional reactivity, but not to cognitive empathy. The results on the ESCQ are moderately correlated with the results on the cognitive empathy measure and low with the results on the measure of emotional reactivity. ESCQ, cognitive empathy and emotional reactivity are significantly related to PSB.

To examine whether PSB can be explained by EI and empathy measures, we conducted a hierarchical regression analysis (in which we included variables that are significantly related to PSB, namely ESCQ, cognitive empathy and emotional reactivity). In accordance with Akamatsu and Gherghel’s model [3], we introduced ESCQ as a predictor measure in the first step of the analysis, and empathy measures in the second step. Table 3 shows the results of the analysis. All predictors were significant in the prediction of PSB and together explain 25% of the variance of PSB.

To test whether empathy (emotional reactivity and cognitive empathy) mediated the relationship between self-assessed EI and PSB, we conducted a mediation analysis using a bootstrapping procedure with 5000 resamples (Table 4). We included gender as covariate. We tested our hypotheses using PROCESS Macro (Model 4) in SPSS [51]. All continuous variables were standardised before the analysis.

**Figure 1:**
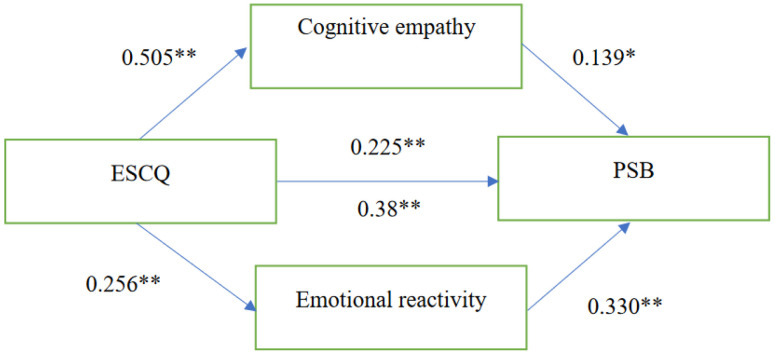
The mediation model: emotional reactivity and cognitive empathy mediate the relationship between self-assessed EI and PSB. Note. *p* < 0.01 **, *p* < 0.05 *.

The results showed (Figure 1) that the significant effect of the self-assessed EI on PSB (β = 0.39, *p* = 0.0001) became weaker/less significant (β = 0.24, *p* = 0.0003) after including emotional empathy and cognitive empathy in the model. Moreover, the indirect effect of both empathies was significant. For cognitive empathy, indirect effect = 0.07, 95% CI [0.082, 0.139], and for emotional empathy, indirect effect = 0.08 95% CI [0.042, 0.138].

## 4. Discussion

The results showed that EI tests intended to measure abilities to understand (TAE) and manage emotions (TUE) are not related to PSB. This relationship has not been investigated much so far. Although we expected that students who better understand verbal emotional content (higher TAE) and better manage their own and other people’s emotions (higher TUE) would show more PSB, this was not the case. Such results confirm the view that ability EI is only a potential that may or may not be used for prosocial purposes [3,49]. On the other side, self-assessed EI is related to PSB, which means that students who perceive themselves to be more effective in solving problems related to emotions are more inclined to PSB. This is consistent with other research on adolescent [8,35,52], college student [5,36] and adult samples [53], but also with the theory according to which EI contributes to a person’s adaptive functions [54]. Given that, according to theory, EI abilities should contribute to the explanation of PSB, the question arises as to why self-reports of EI appear to be important in this context, while EI tests are not. According to our results, for the prediction of PSB, it is important how individuals perceive themselves, that is, how they evaluate their emotional abilities, and not what the actual level of these abilities is. People who perceive themselves as a person for whom emotions are important, who assess that they successfully deal with them and use them in a way that contributes to their well-being and the well-being of the people around them, more often behave prosocially. On the other hand, the way a person solves real problems related to emotions is not related to PSB. What could characterise people who evaluate themselves as more emotionally competent could be an interest in the emotional aspect of life that makes them more sensitive to the problems of others and more willing to help. People who can actually solve problems related to emotions do not have to be interested in it or use their abilities for prosocial purposes. However, it is important to emphasise that we did not measure actual PSB, but we also used assessments of PSB. It would be interesting in future investigations to examine the correlation between EI ability and actual PSB (for example, using the dictator game). Furthermore, it is important to note, when considering the relationship between self-assessed EI and PSB, that the correlation between the same types of measures (self-reports) could be partially based on the method variance. In the case of the relationship between ability EI and PSB, this does not happen.

In our research, the connection between empathy, its emotional and cognitive aspects, and PSB was confirmed, which is in line with the research mentioned in the introduction [29,30,31]. Both of these variables contribute significantly to explaining the variance of PSB, along with self-assessed EI. Those three variables in total explain 25% of students’ PSB. Furthermore, mediation analysis showed that both empathy variables (emotional reactivity and cognitive empathy) are partial mediators of the relationship between self-assessed EI and PSB. This means that people with higher self-estimated EI behave prosocially more often because they experience empathy to a greater extent, both cognitively and emotionally. Akamatsu and Ghelgher [3] proposed a model according to which the presence of empathy in a person leads an emotionally intelligent person to PSB, and the presence of Machiavellianism to antisocial behaviour. We could say that our results confirm part of that model.

In this research, we applied three different measures of EI (two tests and one self-report measure), and their relations with measures of empathy provide some interesting insights. In previous research, EI tests were generally in low correlation with self-assessments of EI [55] and empathy [33], and this was also shown in this study. It is interesting that none of the EI tests in this research is related to cognitive empathy, which as a construct overlaps more with EI compared to emotional reactivity. It seems that people are not good at assessing their capacities, which is in line with many investigations on that topic [33]. However, this result should be further investigated. Correlation of the TAE with emotional reactivity points to the conclusion that students who understand verbal emotional content better are able to experience and react to other people’s emotions to a greater extent. This result may indicate that experiencing emotions is related to their understanding, that is, that we will better understand those emotional contents that we ourselves have experienced. Correlation of TUE with emotional reactivity suggests that emotional reactivity helps us respond more successfully to one’s own needs and the needs of other people, as well as to the needs in situations where intense emotions are experienced. A possible explanation for this relationship is that both of these characteristics have an underlying sensitivity to other people’s emotions. TUE scores (managing emotions), but not TAE, are related to self-assessed EI (ESCQ). Students who scored higher on the TUE rated themselves as more emotionally intelligent, but the correlation is quite low. However, the ability measured by this test seems to be more related to the perception of one’s emotional abilities compared to the ability measured by the TAE. Self-assessed EI is moderately and positively related to cognitive empathy, and low to emotional reactivity and PSB. Correlations of self-assessed EI and empathy are expected given that both are measured by self-reports and are related to a person’s emotional functioning. It can be observed that self-assessed EI is more related to cognitive empathy (although it is also related to emotional reactivity), and EI assessed by tests is related to emotional reactivity (and it is not related to cognitive empathy at all). A possible reason for different patterns of association of two types of EI (ability and self-assessed) with the two aspects of empathy (cognitive and emotional) could lie in the social desirability of the responding. Tests results are generally not liable to socially desirable responding, as participants are asked to provide a correct answer, rather than an assessment of their own functioning. It is possible that it is more difficult to read what would be a socially desirable answer in items of an emotional reactivity measure, so the effect of social desirability is smaller, and the results are more related to the performance of the participants in the test. The opposite could be the reason why the correlation of the EI questionnaire is higher with the cognitive empathy scale compared to the emotional reactivity scale. Both measures are self-reports and likely more susceptible to socially desirable responding.

However, this research has certain shortcomings that may affect the results. Research was conducted on a convenient sample of students, though because the sample consisted of college students of various profiles, we have no reason to believe that there are differences in the research variables between our students and other students in Croatia. Nevertheless, a sample with which we could control to a greater extent the influence of sampling on the results would certainly allow their greater generalisation. Another related limitation is the gender-biased sample, the effect of which we tried to control in the analyses. However, future research should investigate the relationships between EI, empathy and PSB in a more gender-balanced sample. Furthermore, the fact that the research was conducted in the classroom presents a strength in this study because participant self-selection is lower in the classroom than in online research (which have been prevalent in recent years). Furthermore, the self-report measures that were used as measures of empathy and PSB also have their own, already mentioned, limitation, such as socially desirable responding and the (in)ability to objectively assess one’s own characteristics. Finally, our design is of the correlational type, and it is not possible to infer cause-and-effect relationships.

In future research, it would be useful to use some other measures of empathy, such as physiological measures, and of PSB, such as assessments of other people or naturalistic observation [56]. In addition, measures of PSB that are relevant for different situations in different contexts, such as schools, organisations and medical care, could be used. Data from such investigations would provide information for creating educational programmes for enhancing PSB in a specific context.

## 5. Conclusions

The conducted research partially confirmed our hypotheses about the role of self-assessed EI and empathy in the prediction of PSB. The hypothesised contribution of EI abilities measured by tests to prediction of PBS was not confirmed. These results indicate the importance of emotional knowledge training so that people can develop a sense of emotional self-efficacy, because this helps them to be more open to other people’s needs. In addition, it is important to encourage the development of empathy in children and young people, so that they direct their emotional competences to socially desirable and prosocial purposes.

## Figures and Tables

**Table 1:** Descriptive statistics and Cronbach’s alphas of the variables.

Variable	M	SD	Min	Max	Theoretical Range	α
TAE	67.63	7.84	35	83	0–100	0.78
TUE	56.02	10.82	17	82	0–104	0.76
ESCQ	158.35	18.14	95	212	45–225	0.91
Cognitive Empathy	12.44	4.20	3	22	0–22	0.82
Emotional Reactivity	11.92	4.24	1	22	0–22	0.73
PSB	47.22	9.20	17	68	0–68	0.85

Note: TAE—Analysis of Emotions Test; TUE—Emotion Management Test; ESCQ—Emotional Skills and Competence Questionnaire; α—Cronbach alpha coefficient.

**Table 2:** Intercorrelations of the variables (N = 299).

	1	2	3	4	5	6
1 Gender	-					
2 TAE	0.19 **	-				
3 TUE	0.33 **	0.44 **	-			
4 ESCQ	0.07	0.10	0.16 **	-		
5 Cognitive Empathy	0.22 **	0.07	0.09	0.52 **	-	
6 Emotional Reactivity	0.39 **	0.14 *	0.27 **	0.27 **	0.31 **	-
7 PSB	0.08	−0.05	0.05	0.38 **	0.33 **	0.40 **

Note: TAE—Analysis of Emotions Test; TUE—Emotion Management Test; ESCQ—Emotional Skills and Competence Questionnaire; *p* < 0.01—**, *p* < 0.05—*.

**Table 3:** Explanation of PSB: results of hierarchical regression analysis.

	*b*	*SE*	β	95% CI		*p*
				*LL*	*UL*	
Model 1						
Sex	1.64	1.17	0.08	−0.64	3.94	0.161
R^2^ = 0.01						
Model 2						
Sex	1.09	1.09	0.06	−1.04	3.23	0.314
ESCQ	0.19	0.03	0.38	0.14	0.25	0.000
		R^2^ = 0.15 ΔR^2^ = 0.14				
Model 3						
Sex	−1.77	1.11	−0.09	−3.96	0.43	0.114
ESCQ	0.12	0.03	0.24	0.06	0.18	0.000
Cognitive Empathy	0.27	0.13	0.13	0.01	0.54	0.042
Emotional Reactivity	0.65	0.12	0.30	0.41	0.88	0.000
		R^2^ = 0.26 ΔR^2^ = 0.11				

Note. *b* = unstandardised regression weights; *SE* = standard error; β = unstandardised regression weights; CI = confidence interval; *LL* = lower limit; *UL* = upper limit; R^2^ = coefficient of determination; significant at the *p* < 0.05 level.

**Table 4:** Regression coefficients, standard errors, and effects for the hypothetical mediation model (cognitive empathy and emotional reactivity as mediators).

	β	*SE*	95% CI	
			Lower	Upper
Total effect	0.38	0.05	0.2	0.49
Direct effect	0.23	0.06	0.11	0.35
Indirect effect Cognitive Empathy	0.07	0.03	0.01	0.14
Indirect effect Emotional Empathy	0.08	0.02	0.04	0.13

Note. β = unstandardised regression weights; *SE* = standard error; CI = confidence interval.

## Data Availability

Publicly available datasets were analysed in this study. These data can be found here: https://repozitorij.ffos.hr/islandora/object/ffos%3A6270 (accessed on 1 April 2023).
